# Relationships between Rodent White Adipose Fat Pads and Human White Adipose Fat Depots

**DOI:** 10.3389/fnut.2016.00010

**Published:** 2016-04-19

**Authors:** Daniella E. Chusyd, Donghai Wang, Derek M. Huffman, Tim R. Nagy

**Affiliations:** ^1^Department of Nutrition Science, University of Alabama at Birmingham, Birmingham, AL, USA; ^2^Department of Molecular Pharmacology, Albert Einstein College of Medicine, Bronx, NY, USA; ^3^Department of Medicine, Albert Einstein College of Medicine, Bronx, NY, USA

**Keywords:** rodents, humans, obesity, fat pads, fat depot, fat distribution

## Abstract

The objective of this review was to compare and contrast the physiological and metabolic profiles of rodent white adipose fat pads with white adipose fat depots in humans. Human fat distribution and its metabolic consequences have received extensive attention, but much of what has been tested in translational research has relied heavily on rodents. Unfortunately, the validity of using rodent fat pads as a model of human adiposity has received less attention. There is a surprisingly lack of studies demonstrating an analogous relationship between rodent and human adiposity on obesity-related comorbidities. Therefore, we aimed to compare known similarities and disparities in terms of white adipose tissue (WAT) development and distribution, sexual dimorphism, weight loss, adipokine secretion, and aging. While the literature supports the notion that many similarities exist between rodents and humans, notable differences emerge related to fat deposition and function of WAT. Thus, further research is warranted to more carefully define the strengths and limitations of rodent WAT as a model for humans, with a particular emphasis on comparable fat depots, such as mesenteric fat.

## Introduction

Obesity has largely been defined by a body mass index (BMI) >30 kg/m^2^, or better still, a body fat percentage >25% in males and >35% in females (1). However, from a physiological standpoint, evidence indicates that body fat distribution, irrespective of BMI and/or body fat percentage, most strongly predicts risk of obesity-related diseases and complications (2). Risk can further be stratified by fat distribution, as individuals with a higher waist-to-hip ratio suffer disproportionately from obesity-related metabolic dysfunction (3). Indeed, individuals with gynoid obesity, characterized by subcutaneous fat in the gluteofemoral region, have minimal risk of developing metabolic dysfunction (3, 4); whereas individuals with the so-called android obesity, which is characterized by visceral fat accretion, suffer a greater risk of metabolic dysfunction (4, 5).

The metabolic consequences of body fat and its distribution have received extensive attention in the literature. As rodents are by far the most commonly used pre-clinical model of human obesity (6–9), further validation of important commonalities and differences between rodent and humans are needed. Specifically, investigators should seriously consider to what extent their experimental approach and findings are translational. For instance, rodent adipose tissue deposition is strikingly dissimilar to humans, and adipocytes in these depots display metabolic heterogeneity and are intrinsically different within a species. As such, further research needs to focus on how specific rodent fat pads correspond, if at all, with fat depots in humans. To our knowledge, only two studies have compared gene expression in mouse fat pads to gene expression in analogous human fat depots (10, 11). Thus, given the general lack of information or discussion on this highly relevant topic, a systemic review of the literature in our view, is warranted.

## Fat Depots Versus Fat Pads: Anatomical Considerations

**Figure 1:**
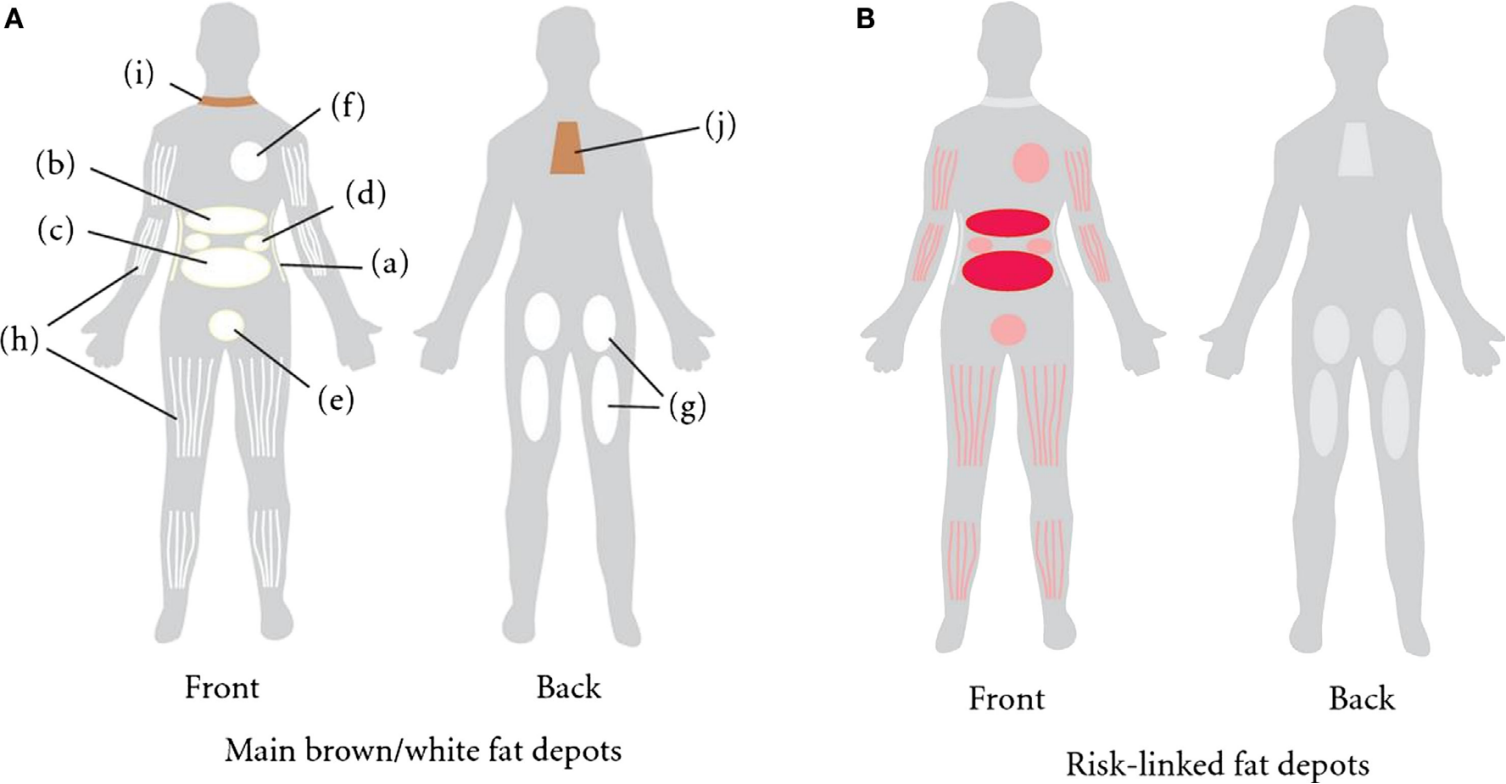
**(A)** Distribution of fat depots in the human. The white adipose tissue (WAT) is found in subcutaneous adipose tissue (SAT) abdominally (a), gluteofemorally (g), and intramuscularly (h). WAT is also found in the visceral adipose tissue (VAT). The visceral depots are omental (b), mesenteric (c), retroperitoneal (d), gonadal (e), and pericardial (f). Brown adipose tissue is found supraclavicularly (i) and in the subscapular region (j). **(B)** Those fat depots linked to increased risk of developing obesity-related morbidities and mortality are colored in red (13).

There are three main regional human anatomical fat depots: intra-abdominal, upper-body/abdominal subcutaneous, and lower body subcutaneous (Figure 1A) (12). Intra-abdominal refers to visceral adipose tissue (VAT), which surrounds the inner organs (13). VAT can further be divided into omental, mesenteric, retroperitoneal, gonadal, and pericardial. The upper-body subcutaneous adipose tissue (SAT) can be categorized depending on if it is situated superficial or deep to the fascia superficialis. The adipose tissue below the fascia is the deep subcutaneous adipose tissue (DSAT) compartment, whereas adipose tissue located superficially to this fascia is the superficial subcutaneous adipose tissue (SSAT) compartment (14, 15). Though SAT is distributed throughout the human body, the main depots are in the abdomen, buttocks, and thighs (16). The buttocks and thighs make up the lower body SAT and are termed the gluteofemoral depot (12, 13).

**Figure 2:**
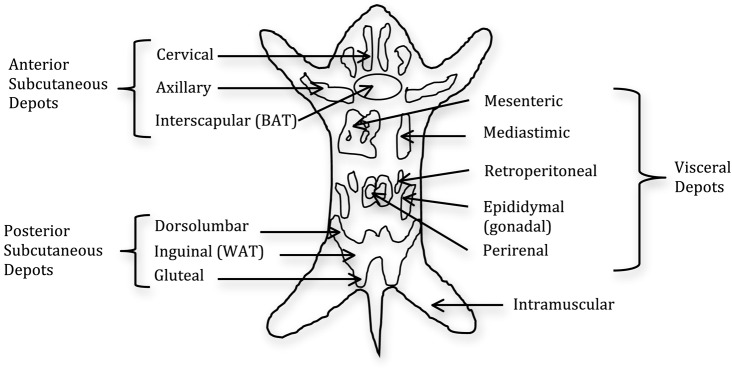
**Distribution of fat pads in the mouse**. The fat is composed of two subcutaneous pads and several visceral pads. The main white adipose tissue (WAT) pads are the inguinal and epididymal, with the latter being the most frequent dissected pad. Brown adipose tissue (BAT) is distributed throughout the fat pads with the main BAT depot in the interscapular region (18).

Like humans, adipose tissue in rodents is a multi-depot organ (Figure 2), but unlike humans, which have two main subcutaneous depots located in the abdominal and gluteofemoral region, rodents have two main subcutaneous pads located anteriorly and posteriorly. The anterior pad is located between the scapulae, descending from the neck to the axillae (17), while, the posterior pad, or inguinal fat pad, spreads from the dorsolumbar region to the gluteal region. The inguinal fat pad is comparable in terms of location to the large gluteofemoral subcutaneous depot in humans. Additionally, rodent SAT is separated from dermal adipose tissue by a smooth muscle layer, whereas, in humans, the SAT is continuous with dermal adipose tissue (17). Furthermore, there has been no evidence to our knowledge of multiple subcutaneous layers in rodents, such as is the case in humans.

Rodents harbor visceral fat pads in the perigonadal region, known as epididymal in males and periovarian in females, as well as retroperitoneal fat pads located on the kidneys, and the mesenteric fat pad located alongside the intestinal tract. The mesenteric fat pad is widely touted as the most analogous to human intra-abdominal adipose tissue both in its location and biology, because it has access to the portal vein. However, this depot is not well studied in rodents due to limitations in its surgical manipulation and separation from contaminating vessels. The perigonadal fat pads are typically the largest and most readily accessible fat pads and, for these and other reasons, they are the most frequently used in the literature (19–21). However, humans do not harbor a fat depot analogous to these fat pads, which has led to the suggestion that these pads should be considered “peri-visceral” rather than true visceral fat pads. Another difference between the two species is the ambiguity when referencing the omental depot in rodents. Though it is clearly defined and developed in humans, it is less so in rodents, leading some investigators to not reference the omental depot at all (13). However, based on the literature, it seems that the omental depot is present in the mouse, albeit in small quantities, and is similarly connected to the stomach, as it is in humans (22). Thus, although adipose tissue is a multi-depot organ in both humans and rodents, there are anatomical differences that should be taken into consideration.

## Fat Pad and Depot Distribution in Health and Disease

In humans, increased VAT is associated with an increased risk for insulin resistance and dyslipidemias (23), while being an independent risk factor for type II diabetes (24), hypertension (25), and all-cause mortality (26) (Figure 1B). By contrast, SAT is associated with improved or preserved insulin sensitivity, and mitigated risk of developing type II diabetes and other metabolic derangements (27, 28). Alternatively, it has been challenged whether VAT accumulation increases the risk for metabolic dysfunction (29, 30). Some have asserted that abdominal subcutaneous fat plays an independent role in developing an unfavorable metabolic profile (14, 31), dependent on how the subcutaneous fat is distributed among abdominal and gluteofemoral subcutaneous fat depots. In rats and mice, respectively, employing a lipectomy model, whereby the epididymal and retroperitoneal fat pads are surgically removed, improves insulin action, reduces tumorigenesis and improves longevity, independent of confounding factors (32–35). It deserves mentioning that surgically ablating the mesenteric fat pad in rodents cannot be sufficiently performed with current techniques due to the heavy innervation and vascularization of this tissue. Thus, given the general sentiment that rodent mesenteric fat is most analogous to the human intra-abdominal depot, it is tempting to speculate that the true importance of VAT gleaned from rodent lipectomy studies, has if anything, underestimated the “true” hazards associated with visceral adiposity. By contrast, some studies have shown that removal of omental fat in obese men and women in conjunction with gastric bypass surgery provided no added benefit to metabolic endpoints (36, 37). However, unlike rodent studies, where ~75% of visceral fat was removed, the omentum in obese subjects likely accounts for a small fraction (<10%) of total visceral adiposity, an amount that may have been insufficient to observe meaningful benefits.

Mechanistically, the benefits associated with SAT may be attributed to its ability to act as a metabolic “sink” to buffer against the daily influx of nutrients by providing long-term energy storage (38), thereby protecting against ectopic fat deposition and associated lipotoxicity (3). Indeed, gluteofemoral fat in humans has been shown to have a protective role, such that it is independently associated with lower triglyceride levels (39), and higher concentration of high-density lipoprotein cholesterol (40, 41). Studies have shown that femoral fat is associated with an elevated adipose tissue lipoprotein lipase activity (40, 42). Though the mechanisms responsible for depot differences in metabolic profiles still remain unclear, speculatively, gluteofemoral fat secretes more beneficial adipokines, as supported by studies in rodents (43), thereby producing less pro-inflammatory molecules compared to VAT (3). Indeed, it is widely believed that the deleterious effects of elevated VAT can be attributed in part to an enhanced secretion of pro-inflammatory cytokines and the release of FFA, which have direct portal access to the liver (41, 44).

In humans, Jensen et al. (5) demonstrated that in moderately obese women, body fat distribution is predictive of FFA dysfunction, such that upper body obese women, but not lower body obese women, had an increased adipose tissue FFA release, when normalized for lean body mass. Furthermore, upper body obese women released almost twice the amount of calories as lower body obese individuals. Speculatively, the differences in metabolic function between these two states could also be attributed to adipocyte hypertrophy, which occurred in the upper body obese women during weight gain, as opposed to hyperplasia, which was observed in the lower body obese. Therefore, the adipocytes in the lower body obese were still of normal size with more restrained lipolysis, while the upper body adipocytes were enlarged with increased lipolysis. Importantly, many of the hazardous metabolic sequelae secreted from adipose tissue have been linked to enlarged hypoxic adipocytes (45, 46).

In rodents, surgically transplanting subcutaneous fat has demonstrated either no deleterious effect or even proven beneficial to the recipient in some reports. By contrast, when subcutaneous fat was removed from mice, there was a significant increase in serum triglycerides and basal insulinemic index, which implies a quantitative role for subcutaneous fat acting as a metabolic “sink” (9, 47). Likewise, subcutaneous, but not visceral fat transplants, improved glucose metabolism in mice, particularly when they were placed in the viscera (43). It should be noted that large-volume liposuction (~10 kg) of subcutaneous fat in humans neither improved nor harmed the metabolic (48) or cardiovascular risk profile (49). Importantly, a redistribution of fat stores is a hallmark of aging and is characterized by a depletion of subcutaneous fat stores in older rodents and humans, which contributes to a simultaneous expansion of visceral and ectopic fat stores in sites such as liver, pancreas, bone, and skeletal muscle (50). Thus, taken together, these observations suggest that subcutaneous fat may be beneficial in both rodents and humans, not only via its own secretions but also by mitigating the accretion of fat in other more harmful sites with obesity and aging, including visceral and ectopic stores.

Beyond the contribution of VAT *per se*, an emerging association with specific sites of SAT have been linked with increased metabolic dysfunction in humans (30, 31, 51). As previously stated, subcutaneous fat is divided into two layers: superficial and deep (14, 15). Johnson et al. (15) investigated abdominal adipose tissue in obese women and determined that the area of DSAT was highly correlated with the area of VAT. Similarly, Kelley et al. (52) examined both lean and obese men and women, and found that DSAT and VAT were both strongly correlated with glucose, insulin, HDL, and triglycerides, whereas SSAT had a much weaker association, and more closely mirrored gluteofemoral SAT. Smith et al. (14) found that DSAT was correlated with VAT, as well as with fasting insulin levels. Because DSAT follows a pattern more associated with VAT, and SSAT parallels gluteofemoral adiposity, the location of adipose tissue sampling, either above or below the fascia, needs to be taken into account as these conclusions support the hypothesis that SAT is heterogeneous, having different physiological effects depending on depot location. Rodents do not harbor fat pads that are clearly analogous to the DSAT layer, though it is possible that the so-called peri-visceral fat pads (gonadal, retroperitoneal), which tracks with mesenteric fat mass, may be relevant candidates to explore in future studies.

## Sexual Dimorphism in Adipose Tissue Regulation and Function

It has been recognized that the development of adipose depots during positive energy balance differs according to sex. Independent of BMI, women typically carry 10% more body fat than their male counterparts (53, 54). Demerath et al. (55) found that women typically have greater total body fat than men. Additionally, compared to men, women have greater SAT in the abdominal and gluteofemoral depots, independent of total body fat (55, 56).

Not only does SAT area and volume differ between sexes, but also the spatial distribution of SAT. Regardless of race, women tend to harbor greater SAT in the lower abdomen, and in general, have lower visceral content than men (55, 56). One explanation for the increase in subcutaneous adiposity in women may be attributed to preferential increases in SSAT rather than in DSAT. The area of the deep subcutaneous depot is inversely associated with fasting insulin levels (14) and, in general, women have lower visceral content than men (55, 56). This may partially contribute to why, on average, pre-menopausal women tend to have healthier metabolic risk profiles compared to men, irrespective of total fat content. Nevertheless, it is acknowledged that additional factors, such as reproductive hormones and differential racial responses, influence both fat quantity and distribution.

As stated previously, women have more SAT than men, while men have more VAT than women (57). Thus, it is not surprising that leptin is a better correlate for body fat in females (58), as SAT secretes more leptin than VAT. By contrast, insulin is a better correlate for body fat in males (58), as insulin is more related to VAT than SAT (57). Because of the aforementioned sex differences in fat deposition, and their relation with various co-morbidities, it is important to consider whether these sex differences similarly exist in rodents, in order to promote their pre-clinical value as a guide for relevant translational research.

To date, the sexual dimorphism seen in humans is less documented in rodents (59, 60), although some inferences have been made. For example, similar to humans, female rats have higher plasma leptin levels compared to male rats (58), independent of body composition differences (61). Consistent with these observations is the brain sensitivity to these hormones, with male rats demonstrating greater central sensitivity to insulin, while females were more responsive to leptin (58).

Similar to humans, female rodents harbor greater fat mass compared to their male counterparts, but remain more insulin sensitive. Macotela et al. (60) showed that isolated adipocytes from subcutaneous fat of female mice were more insulin sensitive than male-derived subcutaneous fat. However, when further examining inter-depot insulin sensitivity in female mice, the periovarian fat pad proved to be more insulin sensitive than the inguinal fat pad (60), a finding that is at odds with data from humans, where the gluteofemoral depot has proven to be the most sensitive SAT in females (62), and is also more insulin sensitive than the VAT (63).

An additional similarity between the species is spatial patterning of the adipose tissue with changes in sex hormones. Postmenopausal women preferentially redistribute adipose tissue from the gluteofemoral region to the abdomen, which mirrors the accrual of visceral fat observed with aging in men. Likewise, Grove et al. (59) demonstrated that ovariectomized female mice demonstrated a significant increase in adiposity similar to adipose tissue accumulation patterning in males, including an increase in total and visceral fat. However, whether ovariectomizing young rodents truly recapitulates female menopause in humans, which occurs during late-middle age, is debatable, and thus should be interpreted with caution.

Though similarities exist, other notable differences in regards to sex differences have been observed between species. One example is the fat pad composition and location associated with sex hormones (i.e., estrogen and testosterone) in rodents. For instance, the inguinal white adipose tissue (WAT) of female mice contains mammary glands, while the gonadal fat pad is situated near the reproductive tissue. This is not the case in humans, where the mammary glands are located in the breast tissue. Furthermore, unlike their male counterparts, several strains of female mice are relatively resistant to obesity on a high-fat diet (64, 65), but this protection can be removed by ovariectomizing the animal (66, 67). Thus, while fat patterning in adult females seems to be influenced strongly by reproductive hormones, overt protection against weight gain and obesity does not seem to be a shared characteristic between female rodents and humans.

## Weight Loss

Regardless of sex, obese individuals undergoing weight loss preferentially decrease their VAT compared to SAT (68). Indeed, significant volumes of VAT are lost when subjected to caloric restriction (CR) and/or a physical activity intervention (69). Additionally, when SAT is mobilized in obese individuals, there is a greater increase in lipid mobilization in the abdominal region compared to the gluteofemoral region (8, 70, 71), which shows only a minimal change in mobilization (71). This preferential reduction in abdominal adipose tissue is consistent with the observation that adipocytes from the omental and mesenteric depots are more lipolytically active, when compared to adipocytes found in the gluteofemoral region (72–74).

When male rodents are subjected to CR or leptin treatment, an examination of weight loss effects on adipose tissue volume and spatial distribution (8, 75) reveals a preferential reduction in VAT compared with other adipose depots (76), while some have shown that male rodents lose both VAT and SAT (77). Similarly, female mice tend to conserve their SAT by preferentially reducing their visceral fat stores (77). This would be somewhat analogous to human studies, where both men and women reduce their visceral fat stores, with a greater extent occurring in men compared to women (78, 79).

Furthermore, as the majority of people who undergo diet-induced weight reduction regain the lost weight over time, it is important to determine if the same phenomenon is seen in rodents. Male mice exposed to *ad libitum* feeding following CR-induced weight loss demonstrated gains in both visceral and subcutaneous fat stores. However, female mice did not follow this same pattern, and were less capable of regaining visceral fat after weight loss (77). Speculatively, this may be attributed to reproductive hormones or to sex differences in leptin concentrations. Circulating leptin decreases with CR, but increases with *ad libitum* feeding in male mice, presumably due to increases in adiposity and food intake. In female mice, leptin patterns appear to differ from males. For example, while leptin concentrations are similarly reduced with CR in females (6, 80), they remain reduced after refeeding, as compared to *ad libitum*-fed controls (80), while others have shown leptin levels unchanged in response to either CR or refeeding (77). Irrespective of weight loss and body composition, women have higher levels of leptin compared to men (55), but both obese men and women who undergo CR, proportionately decrease leptin levels with the reduction of total fat, as well as visceral and subcutaneous fat (68). Interestingly, in children, when adjusting for body composition and fat deposition, sex has no independent effect on leptin concentrations (81). In addition, adults attempting to lose weight tend to engage in multiple episodes of weight loss and regain (e.g., weight cycling); thus, it is important to examine the effects of this flux in body composition and fat deposition on leptin concentration in adult humans. Interestingly, the association between leptin concentration and weight cycling holds true for females, but not for males (82). Importantly, the fact that both female and male mice show an increase in fat deposition after CR in a manner reminiscent of humans during weight regain suggests that rodents may be an informative model toward elucidating many sexually dimorphic traits related to fluctuations in energy balance and storage (83).

## Lipolysis

Lipolysis, which is the active breakdown of triglycerides to FFAs and glycerol, has been widely studied in both rodents and humans. Many striking similarities in the regulation of lipolysis exist among species, including stimulation by catecholamines, growth hormone, testosterone, and cortisol (corticosterone in rodents), as well as inhibition by insulin. Lipolysis can be stimulated in rodents and humans under similar physiological contexts, including fasting, exercise, and stress. While, activation of the sympathetic nervous system and subsequent release of epinephrine, norepinephrine, and cortisol represents a major driver of lipolysis in humans (84) and rodents (85), important differences between rodents and humans exist in how some of these pathways drive lipolysis.

Catecholamine-driven lipolysis, which occurs by activating three different β-adrenergic receptor (AR) subtypes, β1, β2, and β3, provides one important example. ARs are coupled to a Gα subunit, and catecholamine induced activation leads to a cascade of events culminating in lipolysis, including increased cAMP by adenylyl cyclase, activation of protein kinase A, and activation of hormone-sensitive lipase, enabling its translocation to the lipid droplets to catalyze the hydrolysis of triglycerides (86). Both β1-AR and β2-AR are ubiquitously expressed in rodents and humans, whereas β3-AR expression is more tissue and species specific, with expression confined to WAT in rodents, while only being marginally expressed in human adipocytes (87). Indeed β3-adrenergic agonists induce a lipolytic response in rodents (88), but fail to significantly stimulate lipolysis in human adipocytes, *in vitro* (89) and *in vivo* (90).

In humans, α2-ARs, as compared to β-ARs, are highly expressed in SAT, and have a greater affinity for catecholamines (91), while this G-coupled protein receptor is absent in rodent adipocytes. In humans, α2-AR acts to inhibit lipolysis by decreasing cAMP levels. Indeed catecholamine activation of α2-ARs in non-obese humans has been shown to partially down regulate lipolysis in SAT (92). Further support of α2-AR regulating lipolysis was gleamed from rodent studies whereby the human α2-AR was expressed in transgenic mice on a β3-AR knockout background. Specifically, these animals exhibited a blunted catecholamine-stimulated lipolysis response and an obese phenotype (93). Therefore, it appears a balance between α2-AR and β-AR is necessary for lipolysis regulation, at least in humans (87).

Historically, the major circulating factors regulating lipolysis in human WAT have been appreciated to be catecholamines, growth hormone, cortisol, testosterone, and ghrelin (94). However, more recently, it has been shown that natriuretic peptide [extensively reviewed in Ref. (95)] induces lipolysis *in vitro* and *in vivo* (96), independently of the catecholamine-AR pathway (97), via a cGMP-dependent mechanism (96). However, natriuretic peptide lipolysis is apparently primate specific. Sengenès and colleagues showed that only primate adipocytes (i.e., humans and macaques) showed natriuretic peptide-induced lipolysis, while this effect was absent in rats, mice, hamsters, and other non-rodent mammals.

In summary, important similarities exist in the biology of rodent and human adipose tissue lipolysis, but as we have discussed, many important distinctions have been identified that warrant consideration when utilizing rodent models. Such differences are apparent in the species specificity of β-AR expression on adipocytes, and the distinct roles of α2-ARs and natriuretic peptide in regulating human, but not rodent adipose tissue lipolysis. Likewise, some lipolytic agents active in rodent adipocytes fail to have similar effects in human adipocytes (91). For example, adrenocorticotropic hormone (ACTH) induces lipolysis in mouse (98) and rat adipocytes (99, 100), respectively, while alpha-melanocyte-stimulating hormone (αMSH) was also shown to modulate murine adipocytes (101). However, neither ACTH or αMSH – induce lipolytic activity in human adipocytes (98, 102). Therefore, it is important to balance and account for these important differences against commonalities in the biology of adipose tissue lipolysis among species.

## Fat Pads and Fat Depots as an Endocrine Organ

Adipose tissue, which is made up of numerous cell types, including pre-adipocytes, adipocytes, T cells, B cells, and stromovascular cells, is now appreciated as an active endocrine organ, capable of secreting numerous humoral factors. Indeed, systemic levels of adipokines, including leptin, interleukin (IL) 1-β, IL-6, and tumor necrosis factor-α (TNF-α), are actively secreted and levels rise with increasing fat mass. On the other hand, adiponectin has been linked to many beneficial effects, but levels correlate negatively with increasing adiposity (103). Because these adipokines have pleiotropic actions, including extensive metabolic effects, it is important to determine if secretion is similar among humans and rodents with respect to depot location.

As mentioned previously, leptin is preferentially secreted in humans from peripheral subcutaneous depots at a higher rate than from VAT (103, 104), indicating that subcutaneous fat is a chief source of leptin production. Likewise, leptin expression was shown to be greater in rat inguinal as compared to epididymal fat pads (105). IL-6 is another important cytokine that is produced in significant amounts from adipose tissue. Mohamed-Ali et al. (106) showed that SAT is capable of producing IL-6, while Fontana et al. (103) demonstrated that IL-6 secretion is greater from VAT than from SAT in obese individuals (103). Furthermore, IL-6 levels are elevated in middle-aged men with visceral adiposity, as compared to lean men, and adjusting for visceral fat accounts for the rise in IL-6 with visceral obesity (107). Other fat-derived factors, such as TNF-α, show similar secretion patterns in VAT and SAT in humans (103), but adipose-derived TNF-α has been suspected to act in more an autocrine/paracrine rather than endocrine manner.

The data are less clear for adiponectin secretion from different human depots. Phillips et al. (108) showed that SAT secretes more adiponectin compared to VAT, while obese individuals demonstrate impaired total adiponectin secretion from SAT, but not from VAT depots (109). Meanwhile, some have reported no significant difference in total adiponectin secretion between subcutaneous and visceral adipocytes (103, 109), while others have shown that cultured visceral adipocytes secrete more adiponectin compared to subcutaneous adipocytes (104). Likewise, adiponectin expression from rat epididymal fat pads was significantly greater than that from inguinal fat (105).

In regard to evidence from rodents, a microarray study in isolated epididymal and inguinal fat pads from rats first showed marked differences in these tissues, including increased expression of resistin, angiotensinogen, adiponectin, and PPARγ in epididymal tissue, while inguinal fat demonstrated greater expression of PAI-1 and leptin. Studies have also assessed the effect of surgically removing the epididymal and perinephric fat pads on circulating levels of adipokines. Pitombo et al. (110) showed selective ablation of these fat pads restored insulin action and normalized TNF-α, IL-6, and adiponectin levels. By contrast, surgical removal of visceral fat pads in a male and female mouse model of intestinal cancer fed a high-fat diet led to sexually dimorphic responses. In males, lipectomy reduced adiponectin levels, but did not alter other adipokines, presumably due in part to a compensatory expansion of the mesenteric fat pad (6). By contrast, females had higher circulating levels of adiponectin than males, and while lipectomy reduced circulating levels, they remained at higher concentrations than observed in control males. Furthermore, no compensatory change was observed in mesenteric fat pad mass in females, but leptin levels were significantly elevated, suggesting the expansion of subcutaneous fat stores. Unfortunately, the literature does not provide additional information regarding adipokine secretion in rodent models in relation to specific fat pads.

Finally, almost all studies evaluating circulating levels of adipokines are conducted according to clinical trial protocols, which often require the patient to fast overnight prior to blood sampling. However, measuring levels and expression patterns under basal conditions may be misleading as it is now recognized that nutrients can provoke the expression and production of adipokines from adipose tissue. Indeed, most humans (and rodents) spend the majority of their day in the postprandial state, and as a result, the expression and secretion of adipokines from fat depots and fat pads may be severely underestimated. Einstein et al. (111) first showed that when rats were exposed to hyperglycemia and hyperinsulinemia, expression of several peptides in fat, including resistin, adiponectin, leptin, PAI-1, and angiotensinogen, was markedly increased by 2- to 10-fold in epididymal fat pads, but less dramatically in subcutaneous fat. Similar patterns were also observed when animals were challenged with glucosamine (112) or FFA (113), and some of these responses were further exaggerated in aging rats. Similarly, Kishore et al. (114) showed in humans that adipose-derived factors from subcutaneous fat potentiate PAI-1 secretion from macrophages in response to FFAs. Furthermore, this response is similarly exaggerated in macrophages obtained from middle-aged versus younger-adult subjects (115). Mechanistically, these effects have been linked to increased flux through the hexosamine biosynthetic pathway as well as TLR4 receptors. It is also important to keep in mind that these comparisons, while informative, are made on a per milligram tissue or mRNA basis and do not necessarily account for the absolute fat pad size and, hence, overall contribution to whole body levels *in vivo*. Thus, given the inherent size of the visceral pads in relation to the inguinal tissue in rodents, the contribution of visceral fat to these secreted factors, particularly in response to nutrients, could be quite large. However, the relative contribution of visceral fat-derived cytokine release to the overall nutrient response *in vivo*, has not been carefully evaluated. Nevertheless, based on the current literature, it appears that adipokine secretion patterns in humans are predominantly similar to what has been shown in rodent studies.

## Hormonal and Genetic Factors Governing Fat Pad Development and Expansion

As mentioned, many uncertainties remain regarding the mechanistic underpinnings responsible for the physiological differences among depots. During weight gain, different depots enlarge via hyperplasia, hypertrophy, or both (116), with new adipocytes generating more readily in some depots compared to others. The inter-depot physiological enlargement differences are likely influenced by both extrinsic and intrinsic factors.

Genetic factors have also been shown to influence the distribution of adipose tissue (117–119). The BMI of an individual is highly heritable and can possibly account for as much as 70% of the variance (117). However, C57BL/6 mice, which are inbred, and theoretically should be identical genetically, demonstrate marked variance in body mass, adiposity, and feeding behavior, even when the mice are fed the same diet type (120), perhaps due to epigenetic effects (121).

Although genetic factors have been implicated in fat pad growth and expansion, the genetic underpinnings controlling these processes are not as well understood. A few investigations have been conducted to elucidate the gene(s) that moderate the distribution of adiposity. Recently, Loh et al. (122) showed LRP5 is involved in fat distribution. Individuals with gain-of-function *LRP5* mutations are characterized with increased lower-body fat accumulation, compared to age-, sex-, and BMI-matched controls. However, more attention has focused recently on adipocytes from different depots, which have shown differential gene expression (105, 119, 123) and proliferative capacity (124, 125). When examining the transcripts that differed around a quarter were found to be developmental regulators (126–128), in particular the homeobox (HOX) superfamily of genes. Investigations have now started to actively examine differential HOX gene expression between depots in order to assist in determining the underpinnings of abdominal versus gluteofemoral adiposity (10, 123, 128, 129). A summary of the HOX gene network in rodent and human adipose tissue is provided in Table 1.

**Table 1:** **Expression patterns of the HOX gene network in human fat depots and mouse fat pads**.

	Human		Mouse	

GENE	SubQ	Visceral	SubQ	Visceral
HOXA1^a^				
HOXA3^a^		X		
HOXA4^a^	X	X		
HOXA5^a^	X			
HOXA10^a^				
HOXA11^a^				
HOXB1^a^	X			
HOXB2^a^	X			
HOXB5^a^		X		
HOXB8^a^	X			
HOXB13^a^		X		
HOXC4^a^				
HOXC6^a^	X	X		
HOXC11^a^	X			
HOXA10^b^	X			
HOXC6^b^	X			
HOXA2^c^		X		
HOXA3^c^		X		
HOXA4		X		
HOXA5^c^		X		
HOXA9^c^		X		
HOXA10^c^	X			
HOXB7^c^		X		
HOXB8^c^		X		
HOXC8^c^		X		
HOXC13^c^	X			
IRX2^c^				
HOXA5^d^		X		
HOXC8^d^		X		X
HOXC9	X^f^	X^d^		X^d^
Nr2f1	X		X	
Gpc4^d^		X		X
Thbd^d^	X			X
shox2^d^		X		X
Tbx15^d^	X		X	
En1^d^		X	X	
Sfrp2^d^	X		X	
EN1^e^		X	X	
HOXA2^e^	X			
HOXA4		X		
HOXA5^e^		X		
HOXA9^e^		X		
HOXA10^e^	X			
HOXC6^e^	X			
HOXC8^e^	X			
HOXC10^e,f^	X			

*Data were obtained from Cantille et al. (130)^a^, Vohl et al. (123)^b^, Karastergiou et al. (128)^c^, Gesta et al. (10)^d^, Tchkonia et al. (126)^e^, and Brune et al. (131)^f^*.

In addition to genetic contributors, structural and hormonal regulators have been shown to influence fat distribution. Scherer and colleagues (132) examined extracellular matrix components of adipose tissue, specifically collagen VI, under different metabolic conditions. In the absence of collagen VI, adipocytes were capable of unrestricted expansion, resulting in further lipid storage and a reduction in ectopic fat deposition (132). Interestingly, even with greater fat expansion, under both high-fat diet conditions and on an *ob/ob* background, collagen VI knockouts compared to controls had improved metabolic phenotypes. To determine if a similar relationship between increased collagen VI and metabolic stress existed in humans, Scherer and colleagues investigated an Asian Indian population due to their propensity to be more insulin resistant compared to BMI-matched Caucasians (133). Collagen VI alpha 3 (*col6a3)*, a major adipocyte-derived secretory protein with increased expression during states of metabolic stress in *ob/ob* and *db/db* mice, was compared between Asian Indian and control matched Caucasians. Similar to the rodent models, *col6a3* expression was significantly greater in abdominal and gluteal subcutaneous adipose depots in the investigated Asian Indians (132). Collectively, there is evidence that in terms of collagen VI, there may be similar adipocyte physiology in both rodents and humans that may inhibit expansion of adipose tissue.

Similar to the collagen VI model, overexpression of adiponectin in *ob/ob* mice lead to an increase in adipocyte cell number and, thus, increased adipose tissue mass, specifically SAT (134). However, even with the observed hypertrophy, the unrestricted expansion of adipose tissue associated with elevated adiponectin levels resulted in a major improvement in the overall metabolic phenotype, even in an obese state (134). The authors speculated that the improvement in metabolic parameters was partially attributed to increased PPARγ activity in adipocytes, leading to a redistribution of lipids from ectopic sites to SAT. Likewise, increased adiponectin levels may also play a role in human obesity. Bouatia-Naji and colleagues (135) investigated common single nucleotide polymorphisms (SNPs) in the ACDC adiponectin encoding gene in French Caucasians and concluded that hyperadiponectinemia may be associated with severe obesity.

Adiponectin levels are also increased in growth hormone receptor knockout (GHRKO) mice (136), likely because adiponectin is negatively regulated by GH (137). Similar to the above transgenic models, GHRKO mice are characterized by greater relative amounts of body fat in both males and females, with a disproportionate amount of fat deposition in SAT (138). In spite of harboring greater amounts of adipose tissue, GHRKO mice are metabolically healthy, an effect attributed to their increased adiponectin levels (139). Interestingly, Laron dwarfism, which is a human syndrome characterized by defective GH signaling, is characterized by obesity, in spite of a small stature, but these individuals are protected against type 2 diabetes (140) and have elevated adiponectin levels (141). Thus, humans and rodents may have a similar response to reduced GH/IGF-1 signaling and associated increased levels of adiponectin on fat accretion and patterning as well as glucose metabolism.

Glucocorticoids also influence adipose tissue differentiation, function, and distribution. High levels of glucocorticoids partially contribute to visceral obesity in conjunction with diabetes, hyperlipidemia, and hypertension (142, 143). One mechanism for the production of glucocorticoids is through the enzyme 11-β-hydroxysteroid dehydrogenase type 1 (11β HSD-1). Interestingly, Masuzaki and colleagues (144) created a mouse model overexpressing 11β HSD-1 selectively in adipose tissue that reflected 11β HSD-1 levels observed in adipose tissue from obese humans, who are reported to have increased 11β HSD-1 activity. They observed that a modest increase in 11β HSD-1 activity was sufficient to induce hyperphagia and increased VAT accumulation. Furthermore, the increased VAT accumulation was attributed to significantly greater glucocorticoid receptor alpha isoform expression in mesenteric compared to SAT. In addition, increased corticosterone release into the portal vein of rodents may contribute to the observed rise in portal FFA levels, which parallels the increase in FFA levels in humans characterized by high circulating cortisol and metabolic syndrome (145–148). Collectively, it appears that structural and hormonal factors associated with increases in adipose tissue are largely similar in both humans and rodents. Therefore, rodents appear to be a viable model of human adipose tissue regulation by many common hormonal factors.

## Conclusion

It is understood that not all obese individuals are at the same risk for developing metabolic perturbations and that body fat distribution is an important determinant of obesity-related complications. Individuals with increased upper-body adiposity are disproportionately burdened by obesity-related diseases, compared to lower-body obese individuals. Thus, it is paramount that studies continue to elucidate the pathways linking various adipose pads and depots in relation to health and disease, as well as the mechanistic underpinnings dictating how body fat is distributed in order to answer fundamental questions. Rodents are commonly used to model features of human metabolism and obesity, yet it is unclear to what extent rodent fat pads are a suitable model of human fat depots. Here, we have highlighted examples of both shared and divergent traits among rodent fat pads and human fat depots. Given some of the stark differences in adipose tissue location and function among species, we urge careful consideration in experimental design and interpretation when attempting to draw definitive parallels between rodent fat pads and human fat depots.

## Author Contributions

DC, DW, DH, and TN participated in the writing of this review paper. DC and TN conceived the original idea.

## Disclaimer

The opinions expressed herein are those of the authors and not necessarily those of the NIH or any other organization with which the authors are affiliated.

## Conflict of Interest Statement

The authors declare that the research was conducted in the absence of any commercial or financial relationships that could be construed as a potential conflict of interest.

## Abbreviations

BMI, body mass index; CR, caloric restriction; DSAT, deep subcutaneous adipose tissue; HOX, homeobox; IL-6, interleukin-6; SAT, subcutaneous adipose tissue; SSAT, superficial subcutaneous adipose tissue; TNF-α, tumor necrosis factor-α; VAT, visceral adipose tissue.
